# Pasteur’s Investigations on Rabies

**Published:** 1881-12

**Authors:** 


### Pasteur's Investigations on Rabies.

The Paris correspondent of the *British Medical Journal* writes of M. Pasteur:—

This eminent biologist has made some most important contributions to science, and his name will ever be connected with his ingenious researches on fermentation, and other important discoveries; but he has drifted into a more speculative kind of scientific experiments. As an example of this may be mentioned his recent experiments with the saliva from the mouth of a child with rabies, with which he inoculated rabbits and guinea-pigs. All the animals died, and their blood was found to contain myriads of micro-organisms, which he concluded to be the specific germs that produce hydrophobia. He then performed a second series of experiments, by inoculating other rabbits with the blood of those that had succumbed from the first inoculation. These also died, and their blood was found to contain the same micro organisms. He, however, soon discovered, by further experiments, but this time with the saliva of children who had died from other diseases, that the results were precisely similar to those observed with the saliva of the first child. In pushing his experiments still further, but with the saliva of a healthy adult, he met with the same results and the same germs as in the preceding cases. This rather puzzled the persevering experimenter, but he is not so easily beaten; and if he has not yet discovered the real nature of the virus of rabies, he fancies he has laid his hand on the organ that secretes it. According to him, the virus of the rabies is not secreted by the salivary glands, but by the brain—or rather, the latter is the seat of the malady; and in support of his thesis, he inoculated a small portion of the bulbous extremity of the medulla oblongata of a rabid animal under the cerebral covering of a healthy animal. The latter became rabid. These results were recently communicated to the Academy of Medicine, in a paper read by the general secretary for the learned experimenter, which called forth some trenchant remarks from M. Bechamp, who positively refused to accept the principle on which M. Pasteur has hitherto founded most of his theories, and added that it is not outside the body that one must look for the germs or elements of destruction; but they are to be found in our own body, in the form of microzymes, which are the only cause of all fermentation, and the lowest element to which our organism can be reduced. Nothing daunted, however, M. Pasteur continues his parasitic warfare with unbroken zeal; and, by further experiments with human saliva, he has made the startling discovery that the saliva of a person fasting is venomous, as it contains the same parasites as those found in the saliva of children above described; but that, on the person breaking his fast, his saliva is deprived of the venomous quality, as the parasites are taken into the stomach with the food. All this is terrible to contemplate; and even M. Pasteur was confounded, as the result of his experiment was as awful as it was unexpected. The learned biologist made no attempt to offer any explanation, but said that he would for the present only point to the fact, which, he added, was in itself very suggestive.

